# In Vitro Release Dynamics of Atorvastatin-Loaded Alginate Particles for Enhanced Periodontal Treatment

**DOI:** 10.3390/polym17030427

**Published:** 2025-02-06

**Authors:** Imke Hlawa, Thomas Reske, Oleksandra Chabanovska, Malte Scholz, Praveen Vasudevan, Stefan Oschatz, Niels Grabow, Hermann Lang

**Affiliations:** 1 Department of Operative Dentistry and Periodontology, Rostock University Medical Center, 18057 Rostock, Germany; 2 Institute for Implant Technology and Biomaterials e.V., 18119 Rostock-Warnemünde, Germany; 3 Institute for Biomedical Engineering, Rostock University Medical Center, 18119 Rostock, Germany

**Keywords:** periodontitis therapy, alginate microparticles, controlled drug release, atorvastatin, divinyl sulfone, cross-linking, ultrasonic atomization

## Abstract

Periodontitis is a chronic inflammatory condition of the periodontium, which often leads to tooth loss. Recently, statins have emerged as potent anti-inflammatory agents with pleiotropic effects that can potentially outperform conventional periodontal treatments. However, the clinical application of statins is limited by the lack of suitable drug carriers that fit the periodontal region and provide a controlled local drug release. In this study, we address the critical gap in localized periodontal drug delivery and introduce an ultrasound-assisted technique to encapsulate atorvastatin within alginate microparticles (10–400 µm in diameter)—a simple, scalable, and biocompatible solution. While ultrasound is widely used in polymer synthesis, its application in alginate polymerization remains underexplored. To mimic physiological conditions, particles were incubated in artificial saliva at 37 °C, with drug release being analyzed via high-performance liquid chromatography. A methylcellulose-based hydrogel served as a conventional reference product. Results revealed that alginate particles exhibited at least a 10-fold increase in mean dissolution time compared to the methylcellulose gel, indicating superior stability. Increasing atorvastatin concentration extended the time interval needed for 50% of the drug to be released (t_50%_) from 1 h to 11 h, maintaining the overall drug diffusion level for several days. Further analysis showed that covalent cross-linking of alginate with divinyl sulfone significantly delayed the initial drug release by 3 h (*p* < 0.05) due to the additional molecular stabilization. These findings underscore the utility of ultrasonic atomization for the processing of alginate-based formulations. Given the ease of production, biocompatibility, and small size, successfully fabricated alginate particles represent a promising carrier for delivery of statins or other related drugs in clinical dentistry.

## 1. Introduction

Periodontitis is a global health problem. According to recent estimates, approximately 62% of the global population was affected by moderate periodontitis, while 23.6% suffered from severe periodontitis between 2011 and 2020 [1]. Periodontitis is characterized by progressive inflammatory destruction of the periodontium in response to pathogenic bacteria on the tooth surface. As the inflammation advances into deeper structures, gingival pockets form, and the alveolar bone gradually deteriorates. These processes, collectively known as clinical attachment loss, can ultimately result in tooth loss [2]. However, it is known today that the dysbiotic biofilm and host immune system with a disproportionate response are responsible for the development and progression of periodontitis [3]. Therefore, systemic inflammatory diseases, such as diabetes and cardiovascular disorders, also affect the course of periodontitis [4,5].

While patients with periodontitis are encouraged to optimize their oral hygiene, scaling and root planing are often required as common treatments in dental practice [6]; these processes include cleaning of the tooth and root surface by the dentist using mechanical instruments. Due to the limited visibility and presence of anatomical niches in the tooth, complete removal of the biofilm is unlikely to be achieved. Furthermore, rapid recolonization of tooth surfaces is typically observed within 3–6 months [7]. Consequently, dentists have adopted the use of local pharmacotherapies to enhance treatment outcomes by improving infection control, mitigating tissue destruction, and supporting periodontal regeneration [8]. Local administration within a periodontal pocket is particularly advantageous, as it provides targeted efficacy at the specific site of infection. Additionally, this approach minimizes systemic side effects and preserves beneficial gut microbiota [9]. If drugs offer a delayed release and reproducible kinetics, they are usually referred to as controlled release systems [10]. Such carrier systems must be adjusted to the application site to ensure an optimal therapeutic effect. Otherwise, the administered therapeutic agents may be rapidly washed out by gingival crevicular fluid and saliva [11].

Polymer-based hydrogels and chips have previously demonstrated their utility in the periodontic field. The use of polymer films, fibers, vesicular systems, and pastes has been explored in several clinical trials [12,13]. However, existing systems demonstrate considerable disadvantages. For example, periodontal chips often require manual adjustment to fit the size of the gingival pocket (e.g., 4 × 5 mm for PerioChip^®^) which is a time-consuming process. Pastes have the disadvantage of a very fast and short-time release, while previously used carriers (like ethyl vinyl acetate fibers) were not bio-absorbable and had to be replaced when they were exhausted [14]. Alginate particles appear to be suitable as a periodontal drug carrier as they are biocompatible and easy to apply. Alginates are natural degradable polysaccharide polymers that can be isolated from bacteria and brown seaweed. They consist of linear copolymers with blocks of (1,4)-linked β-d-mannuronate (M) and α-l-guluronate. Adding divalent ions (e.g., Ca^2+^) to an aqueous alginate solution results in a gel structure that allows for the simple generation of alginate particles. Previously, Scholz et al. described a production method for alginate-based carriers for periodontal applications using a dental scaler [15]. Resulting particles demonstrated sizing in a micrometer range. In a clinical setting, such microparticles could fit into any gingival pocket and are likely to optimally disperse. The mucoadhesive properties of the alginate could also lead to a prolonged retention time in the gingival pocket [16].

Although antibiotics or disinfectants are widely used in periodontal therapy, a deeper understanding of disease pathogenesis has shifted the focus toward drugs with anti-inflammatory and host immunomodulatory properties. Statins as a drug class are suggested as novel, highly potent dental therapeutics, especially for periodontitis cases [17]. Statins are primarily used for lowering cholesterol; however, they also exhibit considerable pleiotropic effects, including immunomodulatory, anti-inflammatory, and bone resorption inhibiting properties [18]. In the context of oral health, atorvastatin, a member of the statin class, has demonstrated significant clinical and radiographic improvements in bone defects when used as an adjunct to the non-surgical periodontal treatment of deep pockets compared to instrumentation alone [19,20,21,22]. Moreover, atorvastatin, as an adjunct to instrumentation, has significantly enhanced periodontal regeneration during a clinical trial [23]. In the abovementioned clinical studies, statins were incorporated into hydrogels for periodontal use. Specifically, methylcellulose was repeatedly used as a drug carrier [19]. Methylcellulose is one of the most important commercial ethers of cellulose. However, methylcellulose hydrogels generally have reduced stability in an aqueous environment and lower mechanical properties than alginate [24]; its drawbacks emphasize the need for an alternative drug carrier.

The release of drugs from delivery systems is a complex process. Mathematical models are used to describe the transport mechanisms, which may include drug diffusion and dissolution, as well as osmosis, swelling, erosion, disintegration, and/or degradation of the delivery system [25]. The Korsmeyer–Peppas equation is a pivotal model in understanding the release kinetics of polymeric systems. The equation-derived diffusion exponent defines different transport mechanisms. Case I transport corresponds to Fickian diffusion, where drug release is primarily driven by passive diffusion resulting from concentration gradients. Non-Fickian (anomalous) transport occurs when drug release is influenced by both diffusion and relaxation of the polymer matrix. Case II transport is characterized by swelling and relaxation as the dominant forces of drug release. Finally, Super Case II transport is observed in the case of a significant carrier erosion [26].

The aim of this in vitro study was to develop an alginate-based controlled release system for atorvastatin as a therapeutic application for periodontitis. For this purpose, we optimized our previously reported preparation method for drug-loaded microparticles [15] by improving the aspect of time efficiency. Utilization of the ultrasonic processor instead of dental ultrasonic scaler notably reduced the preparation time. The release kinetics of atorvastatin were investigated in a time-dependent manner under conditions imitating oral cavity (artificial saliva at 37 °C) and were examined by high-performance liquid chromatography (HPLC). Several parameters such as alginate content, particle size, and atorvastatin concentration were varied to identify the most optimal conditions for successful drug release. A conventional hydrogel based on methylcellulose (MC) served as a reference delivery system. Further improvement of the preparation technique was attempted by introducing divinyl sulfone (DVS) as an additional covalent cross-linking agent that was expected to enhance particle stability and, therefore, prolong drug release. The alginate microparticles demonstrated superior properties to methylcellulose gel in terms of prolonged release that was observed over several days. Therefore, we proposed our time-optimized preparation method as a simple, practical option for the encapsulation of periodontal therapeutics designed to provide an extended release—a highly beneficial feature for future clinical treatments.

## 2. Materials and Methods

### 2.1. Drug Incorporation in Alginate Solution

Sodium alginate (Sigma–Aldrich, Darmstadt, Germany) was dissolved in purified H_2_O to prepare 2%, 3%, 4%, 5%, and 6% *w*/*w* solutions that were subsequently subjected to constant stirring using a magnetic stirrer (Heidolph MR 3001, Schwabach, Germany) at 300 rpm for 24 h. Calcium atorvastatin (Sigma–Aldrich, Darmstadt, Germany), provided as a white crystalline powder, was precisely weighted using a precision balance (Denver Instrument, Norfolk, UK) and added to alginate solutions (5 mL) to achieve final concentrations of 1%, 2%, and 4% *w*/*w*, which was followed by a brief stirring with a glass rod. To ensure homogeneity, the mixtures were further subjected to machine-assisted mixing using a SpeedMixer^®^ (DAC 150.1, Farmington Hills, MI, USA) for 5 min at 3500 rpm.

### 2.2. Preparation of a Methylcellulose Hydrogel

As described by Pradeep et al., 4 g of methylcellulose (Sigma–Aldrich, Darmstadt, Germany) was dissolved in 96 g of purified water under constant stirring at 50 °C on a magnetic stirrer (300 rpm) until a homogeneous solution was obtained [27]. To achieve a concentration of 1% atorvastatin, 4.92 g of methylcellulose gel was mixed with 59 mg atorvastatin for 5 min in a SpeedMixer^®^ at 3500 rpm.

### 2.3. Production of Alginate Microparticles with Dental Scaler and Ultrasonic Processor

**Figure 1:**
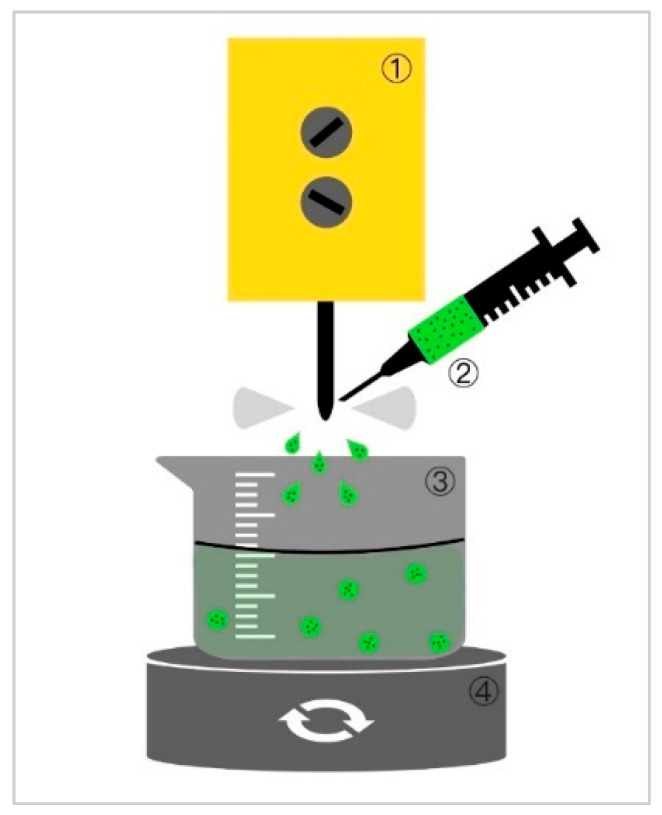
Schematic workflow demonstrating equipment utilized for the production of alginate microparticles. Experimental setup included (**1**) dental scaler or ultrasonic processor, (**2**) syringe with a 20 G needle used for application of alginate–atorvastatin mixture on the tip of the sonotrode, (**3**) beaker with 3% calcium chloride solution, where alginate microparticles were formed and collected, and (**4**) magnetic stirrer to prevent agglomeration of the particles.

Atorvastatin-loaded particles were produced according to the method described by Scholz et al. [15]. The handpiece of a dental ultrasonic device (EMS Mini Piezon, Domat/Ems, Switzerland) or an ultrasonic processor (UP100H, Hielscher, Teltow, Germany) with a sonotrode of size 3 was attached to a stand (Figure 1). A beaker containing 200 mL of 3% calcium chloride solution (Merck, Darmstadt, Germany) was placed on a magnetic stirrer at a distance of 3 cm from the instrument tip. The stirring speed was set to 300 rpm. The dental scaler was set to the maximum power. In the case of the ultrasonic processor, the amplitude was set to 60% and the cycle was set to 100%/s to achieve the fastest spraying (0.05 mL in >2 s). Using a syringe (5 mL; 20 G needle; 0.9 × 40 mm), the alginate–atorvastatin mixture was applied slowly and continuously to the tip. After spraying 5 mL of the mixture, a milky calcium bath with turbidity was observed, which indicated the succesfull dispersion of microparticles in the liquid. No particles could be produced for alginate concentrations of 5% and 6% *w*/*w* due to the high viscosity that prevented the required spraying.

### 2.4. Collection and Size Sorting of Microparticles

**Figure 2:**
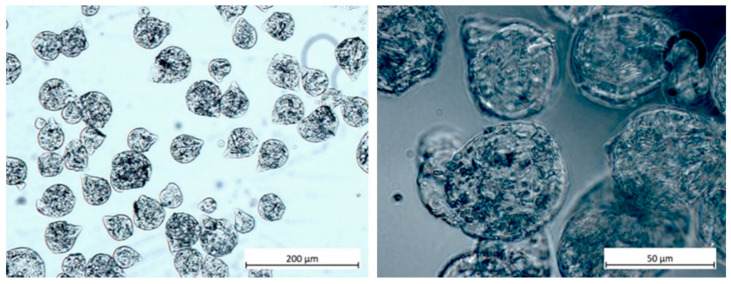
Light microscopy images of atorvastatin-loaded alginate microparticles. Particles with diameters between 10 and 200 µm were produced by an ultrasound processor (left and right images magnified by 10× and 40×, respectively).

The microparticles were collected from the calcium bath and sorted using sieves with mesh sizes of 10 µm (Innovatek, Stammham, Germany), 200 µm, and 400 µm (Labstellar, Ulm, Germany). For this purpose, a 200 mL beaker was placed under a 400-µm sieve and the calcium chloride–particle mixture was poured through. The particles (>400 µm) residing on the sieve were discarded. For the next separation, another 200 mL beaker was placed under a 200-µm sieve, and filtration was repeated. Particles captured on the filter (200–400 µm) were carefully washed with 50 mL of purified water and transferred to centrifuge tubes (15 mL) with a Heidemann spatula. The filtered mixture in the beaker containing particles <200 µm was poured through a 10-µm sieve. Following the same washing steps, collected particles were transferred to Falcon tubes. Two size fractions were obtained: articles with diameters of 10–200 µm and 200–400 µm. All produced particles were stored in a fridge at 5 °C in 10 mL purified water until further use. A light microscope (Scope.A1, AxioCam ICc 1, Carl Zeiss AG, Oberkochen, Germany) was used to observe the particle morphology that appeared round or drop-shaped (Figure 2).

### 2.5. Covalent Cross-Linking with DVS

Based on a protocol of Shimojo et al. [28], 10 mL of alginate–atorvastatin mixture (2% *w*/*w* alginate, 2% *w*/*w* atorvastatin) was mixed with 2.5 mL divinyl sulfone (9.96 M; Sigma-Aldrich, Darmstadt, Germany) with the slow addition of 12.5 mL NaOH (0.1 M; Merck, Darmstadt, Germany). The resulting product was incubated for 4 h at 25 °C and used to generate particles for the ultrasonic method described above. In order to improve the study power to detect a relationship of statistical significance for this investigation, biological replicates were produced by preparing three different alginate–atorvastatin mixtures with DVS and then spraying in three separate calcium chloride baths. Five technical replicates of particles were filtered from each bath and individually subjected to the release analysis. As a reference, three alginate–atorvastatin mixtures (2% *w*/*w* alginate, 2% *w*/*w* atorvastatin) without cross-linker were also prepared to generate replicates accordingly.

### 2.6. Evaluation of Carrier Stability

To determine the stability of the drug carriers, dissolution time (DT) was introduced as a value representing the time interval at which the drug carrier could no longer be macroscopically distinguished from artificial saliva.

### 2.7. Experimental Setup for the Analysis of Release Kinetics

Alginate does not contain any bonds that can be hydrolyzed by human saliva. Degradation is achieved by replacing divalent calcium ions with the monovalent cations sodium or potassium [29]. Since only ions play a role in the degradation of the alginate, the formulation of artificial saliva was simplified (Table 1). The pH was set to 7.

Prepared alginate microparticles were dried on a qualitative filter paper (90-mm diameter, Whatman, Maidstone, UK) for 10 s and transferred to Eppendorf micro reaction vessels (1.5 mL). In the case of methylcellulose, gel loaded with 1% atorvastatin was filled directly into the Eppendorf vessel. Mass of each sample was measured using a precision balance to obtain samples of equal weight. Subsequently, 600 µL of artificial saliva was added to each sample. In order to avoid larger aggregates and ensure a homogenous distribution of the microparticles, the samples were placed on a vortexer (VWR International, Darmstadt, Germany) for 5 s. All samples were stored in an incubator heating cabinet at 37 °C for defined time intervals (0.25, 0.75, 1.5, 3, 6, 10, 17, 33, 57, 81, 105, 129, 153, and 177 h). After each interval, the samples were briefly centrifuged at 1000 rpm for 3 min (Fresco™ 17, Thermo Fisher, Darmstadt, Germany) to sediment the particles or hydrogel. 100 µL of artificial saliva was retrieved for the following HPLC analysis, and the remaining 500 µL was discarded. Then, 600 µL of new artificial saliva was added to the samples, which was followed by a brief vortexing and further incubation at 37 °C until the next time interval, at which the described sampling process was repeated.

### 2.8. High-Performance Liquid Chromatography

A total of 100 µL of artificial saliva collected from each sample was diluted with 900 µL of methanol (99.9%, Rotisolv, Carl Roth, Karlsruhe, Germany). HPLC was performed to determine the released quantity of atorvastatin. Table 2 summarizes the technical properties and settings utilized for HPLC-assisted drug detection. Drug release was expressed as cumulative release (%) over time (h). The total mass contained in each sample was determined as the total released drug content equal to 100%. The time required for 50% and 99.5% of the drug to be released (t_50%_ and t_99.5%_) was calculated arithmetically. To evaluate the stability of the drug carrier, dissolution time (DT) was introduced as a value representing the time interval at which the drug carrier could no longer be macroscopically distinguished from the artificial saliva.

### 2.9. Calculation of the Release Exponent n

The diffusion mechanism of each formulation was described based on the Korsmeyer–Peppas model

M_t_/M = k t*^n^*,

where M_t_/M represents the fraction of released drug at time t, k is the kinetic constant, and *n* is the release exponent. The logarithmic form of the equation,

log(M_t_/M) = *n* log t + log k,

was used for linear regression analysis. The release exponent *n* was determined as the slope of the logarithmic plot in the initial 60% release phase. The calculated values are summarized in Table 3.

### 2.10. Statistical Analysis

Statistical calculations were performed using SPSS (IBM version 29.0.1.1.). The values were given as mean ± standard deviation (SD). Non-parametric variance analyses were performed. The Kruskal–Wallis test was used as a pre-test to compare groups with n > 2. The Mann–Whitney–U test was used for pairwise comparisons of independent groups. *p*-values < 0.05 (Bonferroni adjusted for multiple testing) were considered statistically significant.

## 3. Results

Table 3 gives an overview of the release properties of different formulations based on methylcellulose gel (MC) or alginate carrying atorvastatin (Ator) and prepared using a dental scaler (DS) or an ultrasonic processor (UP) with or without additional cross-linking by DVS.

### 3.1. Comparison of Drug Release from Methylcellulose Gel and Dental Scaler-Generated Particles

**Figure 3:**
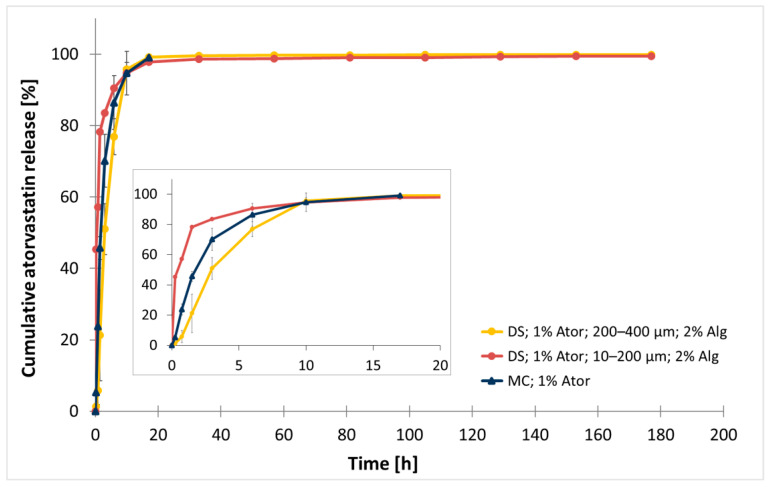
Comparison of normalized cumulative atorvastatin release from different carrier formulations. Methylcellulose gel (MC) served as a conventional carrier reference. Microparticles containing 2% sodium alginate (Alg) were produced by a dental scaler (DS) and divided in two groups based on size (10–200 µm vs. 200–400 µm). All delivery systems contained 1% of atorvastatin (Ator). The data are represented as the mean ± SD, with n = 3 for the MC and large groups and n = 1 for the smaller group.

First, the drug release kinetics of atorvastatin from a conventional drug carrier based on methylcellulose gel were compared to those of alginate-based formulations prepared by a previously described method using a dental scaler (DS) [15]. The MC gel, containing 1% atorvastatin, released 50% of the drug (t_50%_) after 1.78 ± 0.18 h of incubation in artificial saliva, reaching a peak of 99.5% (t_99.5%_) at 12.5 h (Figure 3; Table 3). Complete dissolution of the MC gel was observed after 17 h of incubation, indicating that the gel stability was limited to less than one day.

In contrast, alginate-based formulations with 1% atorvastatin and 2% alginate concentration showed varied drug release profiles depending on particle size. Smaller particles (10–200 µm) released half of the enclosed drug concentration 4 times faster (0.45 h) than the MC gel. On the other hand, larger particles (200–400 µm) exhibited a 2 times slower release compared to MC with t_50%_ of 3.08 ± 0.39 h. Complete drug release (t_99.5%_) was achieved after 169 h for the smaller particles and after 26.44 ± 6.66 h for the larger particles. However, visible agglomeration of the small particles was observed during the release study, potentially affecting their performance.

Notably, alginate-based carriers maintained structural stability for over 177 h, ensuring durability for at least 7 days.

### 3.2. Effects of Sodium Alginate Concentration and Particle Size on Drug Release

For the time-optimized particle preparation method, an ultrasonic processor (UP) was utilized. Several particle formulations with varying alginate concentrations (2%, 3%, or 4%) were analyzed in terms of atorvastatin release over 180 h (7.5 days).

**Figure 4:**
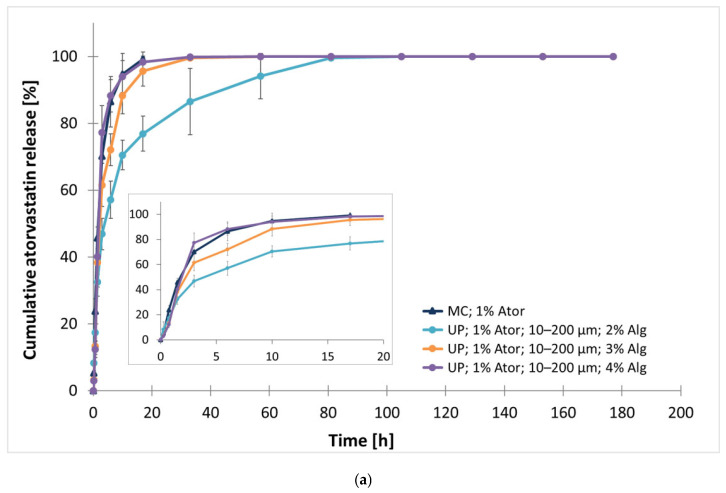

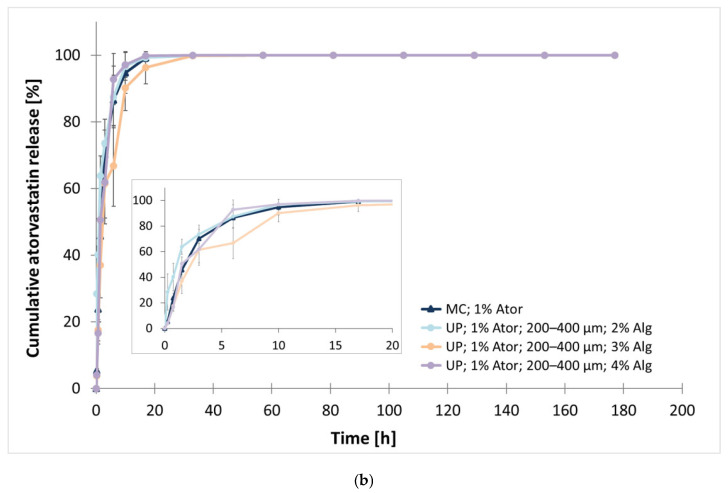
Effect of alginate concentration on normalized cumulative atorvastatin release from microparticles. Particles containing 2%, 3%, or 4% sodium alginate (Alg) were produced by an ultrasonic (US) processor and divided in two groups based on (**a**) smaller (10–200 µm) and (**b**) larger (200–400 µm) particles. All delivery systems contained 1% atorvastatin (Ator). The data are represented as mean ± SD, with n = 3 for MC and n = 5 for the other groups.

In the group of smaller particles (10–200 µm; Figure 4a), particles with 4% alginate reached the t_50%_ value after 1.91 ± 0.21 h of incubation. For 3% alginate content, the t_50%_ slightly increased to 2.24 ± 0.42 h. Particles with the lowest alginate concentration (2%) exhibited significantly prolonged drug release levels, reaching t_50%_ after 4.15 ± 0.98 h, which was twice as long as that of particles with 4% alginate (*p* = 0.008). Notably, small particles produced by UP had a t_50%_ nearly 4 times longer than those produced by DS under similar conditions (1% atorvastatin, 2% alginate) and twice as long as MC gel. The inverse relationship between alginate content and release kinetics was also reflected in the t_99.5%_ value. The shortest t_99.5%_ interval was recorded for the 4% alginate group (19.09 ± 8.38 h), followed by the 3% alginate group (30.82 ± 10.88 h). The 2% alginate group demonstrated the longest release interval (71.35 ± 19.86 h), although notable particle agglomeration in this group could have affected the release kinetics.

The group of larger particles (200–400 µm) presented a comparable release profile when using a higher alginate content (Figure 4b). Particles with 3% alginate exhibited a t_50%_ of 2.58 ± 0.90 h, which was significantly longer than that with a 2% content (1.00 ± 0.27 h, *p* = 0.008). Increasing the concentration to 4% did not significantly extend the t_50%_ (1.94 ± 0.68 h). The 2% group released the drug 4 times faster than the corresponding smaller group and 3 h faster than similar particles produced by a dental scaler. For t_99.5%_ interval, only the 3% formulation achieved a delayed release of encapsulated atorvastatin at 24.10 ± 8.62 h that would surpass the performance of MC gel.

All produced particles retained their structural stability for at least 177 h.

### 3.3. Effect of Atorvastatin Concentration on the Release Profile

**Figure 5:**
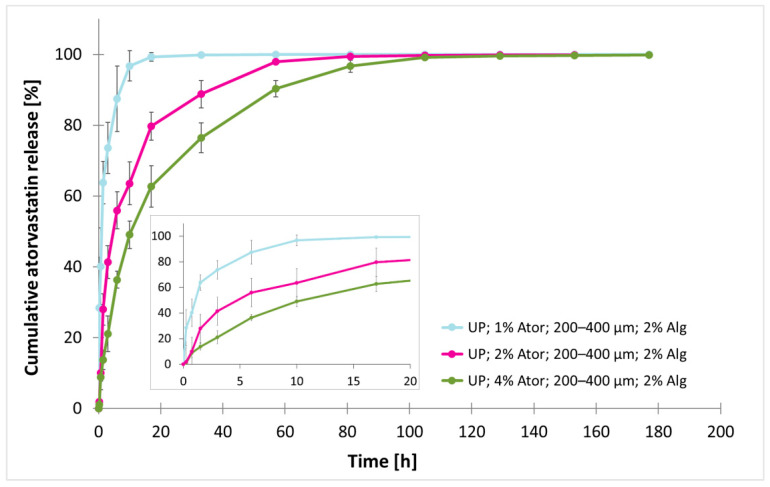
Effect of atorvastatin concentration on normalized cumulative atorvastatin released from microparticles. Particles in the size range of 200–400 µm were produced by an ultrasonic (US) processor and contained 1%, 2%, or 4% atorvastatin (Ator). All formulations contained 2% sodium alginate (Alg). The data are represented as the mean ± SD, with n = 5 for particles with a 1% drug load, n = 3 for particles with a 4% drug load, and n = 15 in particles with a 2% drug load.

To evaluate the loading capacity of the alginate microparticles in relation to the release profile, the concentration of atorvastatin was increased to 2% and 4%. To avoid the possible impact of agglomeration, only particles in the range of 200–400 µm were analyzed. The use of 2% sodium alginate was preferred to provide additional free volume for subsequent drug incorporation. Loaded particles in the size range of 200–400 µm with 2% sodium alginate were subjected to analysis. Figure 5 illustrates that doubling the atorvastatin content to 2% extended the t_50%_ value to 4.97 ± 1.32 h, which was a 5-fold prolongation compared to the 1% drug loading. A further doubling to 4% atorvastatin increased the t_50%_ interval to 10.83 ± 1.07 h. Overall, the time to detect 99.5% of the released drug was recorded at 91.26 h (3.8 days) and 123.35 h (5.1 days) for the 2% and 4% atorvastatin formulations, respectively. Therefore, at least a 7-fold release delay was observed in particles with increased drug load.

### 3.4. Effect of Divinyl Sulfone Covalent Cross-Linking

**Figure 6:**
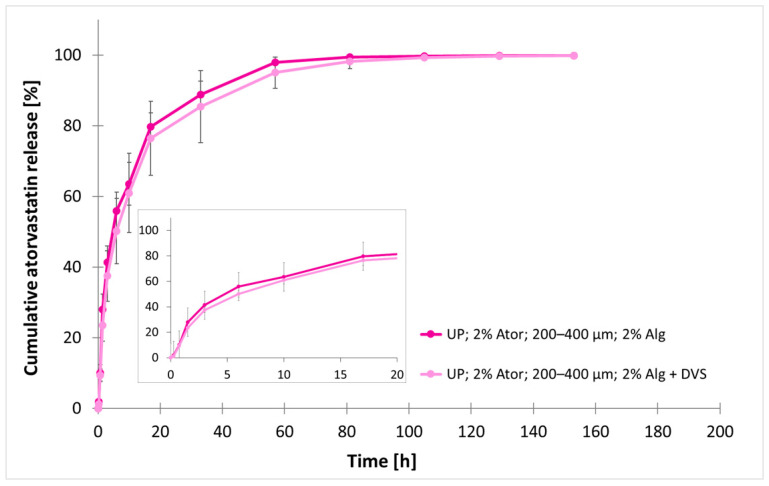
Effect of divinyl sulfone (DVS) addition on normalized cumulative atorvastatin released from microparticles. Particles in the size range of 200–400 µm were produced by an ultrasonic (US) processor and contained 2% atorvastatin (Ator) and 2% sodium alginate (Alg). The data are represented as mean ± SD, with n = 15 for each group.

Divinyl sulfone was introduced to the formulation as a covalent cross-linker to enhance particle stability. For this experiment, atorvastatin concentration was set to 2%, and the release kinetics from the larger group (200–400 µm) were preferably examined to avoid the effect of agglomeration on the release (Figure 6). A significant release delay from 4.97 ± 1.32 h to 7.52 ± 3.08 h was observed under DVS addition (*p* = 0.006). Time to 99.5% release was not affected by DVS cross-linking (t_99.5%_ = 95.84 ± 16.07 h with DVS vs. t_99.5%_ = 91.26 ± 11.19 h without DVS).

### 3.5. Mechanisms of Drug Release for Different Formulations

The Korsmeyer–Peppas model was applied to describe the release mechanism of the presented formulations by means of the calculated release exponent *n* (Table 3). The conventional MC gel demonstrated a Super Case II polymer-controlled transport (*n* > 0.89). DS-derived polymers featured diffusion-controlled transport in the case of small particles (*n* < 0.45) and a strong Super Case II behavior in large counterparts. However, particles produced by UP followed a mixed (anomalous) transport mode (0.45 < *n* < 0.89) when small and mainly diffusion when large (*n* < 0.45). Increasing alginate concentration in UP formulations shifted the release from diffusion to Super Case II. Increasing atorvastatin load resulted in borderline Case II to Super Case II transport (*n* = 0.90). The addition of DVS further favored Super Case II release (*n* = 1.08).

## 4. Discussion

In this study, atorvastatin-loaded alginate microparticles were produced by an ultrasonic method in a time-efficient manner. Drug release was analyzed under simulated physiological conditions (incubation in artificial saliva 37 °C) over several days for particles with different parameters, such as size, content of the main compound, drug load, and addition of covalent cross-linking agent.

In the context of periodontal therapy, many previously developed drug carriers, e.g., non-degradable fibers and films, must be removed by the dentist once the treatment is complete [30,31]. Today, drug delivery systems are primarily designed as biodegradable polymers. Materials like chitosan, pectin, hyaluronic acid, and polyethylene glycol are used to produce micro- and nanoparticles that dissolve naturally and do not require an additional intervention for removal [32]. Therefore, biodegradability of dental therapeutics is essential for both the dentists and patients to ensure a time-efficient treatment. In this respect, calcium cross-linked alginate has shown optimal biocompatibility and degradation properties, leading to its widespread use in medicine as a drug carrier [33]. Several preclinical and in vivo studies utilized alginate for periodontal therapy. Fernandes et al. describes the successful use of alginate particles to deliver growth factor-enriched plasma, which significantly promoted in vivo bone regeneration in periodontal areas [34]. In other studies focused on rats and human cells, alginate-based systems were suggested as a reliable delivery tool for diverse applications in dentistry [35,36,37,38].

The production process for the microparticles described in our study can be defined as ionotropic gelation, which is characterized by the dripping of alginate solution into a salt bath containing divalent ions. It is one of the most commonly used one-step processes [39]. Advantages of this method include easy performance, non-toxic properties, and mild chemical conditions suitable for drug loading. Numerous other methods for the preparation of alginate particles have been outlined in the literature, using physical methods such as spray drying and electro dispersion, as well as physico-chemical methods such as emulsification [40,41,42]. However, physico-chemical techniques are mainly suitable for production in the nanometer range and require sophisticated laboratory equipment. Emulsification is a two-step technique that relies on additional reagents to dissolve particles from the emulsion, which greatly increases the production time [39]. The use of ionic gelation ensures fast and efficient production, offering a convenient method for particle generation. Moreover, the required ingredients are inexpensive, widely available, and highly biocompatible.

Various spray techniques are available for droplet formation such as air-assisted, pressure-based, electrostatic, microfluidic, or nozzle systems [43,44]. However, ultrasonic atomizers have been recognized as a highly advantageous novel tool for microencapsulation compared to conventional methods. In particular, ultrasonic spraying produces more uniform particles in terms of shape and size. The production process requires less energy and offers higher operating speed and industrial scalability [45,46]. At present, the scientific evidence for ultrasound-assisted spraying used to encapsulate drugs in alginate polymers is sharply limited to two studies. Cascone et al. (2012) performed a drug release analysis from alginate microbeads under acidic conditions to imitate stomach environment [47]. Due to the poor drug load, the desired controlled release could not be achieved. However, the insufficient outcome was reasoned by the low molecular mass of the drug and by acid exposure rather than ultrasonic atomization. In our previous report, we utilized a dental scaler as an ultrasonic nozzle for the production of drug-loaded alginate for periodontal therapy [15]. This method was adopted in the current study for microencapsulation of atorvastatin. Although particles produced by dental scaler demonstrated moderate release delay in the larger group (t_50%_ = 3.08 h and t_99.5%_ = 26.44 h), the operating process was generally lacking the aspect of time efficiency. Furthermore, the primary application of dental scalers is in dental hygiene for the removal of plaque and calculus. It is not designed for the production of biomaterials and, therefore, its practical use for microencapsulation is uncertain. The ultrasonic processor operates similarly to a dental scaler but offers significantly greater power, enabling the production of the same quantity of drop-shaped particles within minutes instead of hours.

In past clinical studies, statins for periodontal use were mostly incorporated into methylcellulose hydrogels [19,27,48,49]. Methylcellulose is one of the most important commercial ethers of cellulose [50]. However, major disadvantages of such hydrogels include limited retention, rapid dilution by saliva, and inconsistent drug release. In our static experiments, the methylcellulose gel was dissolved after 17 h of incubation in artificial saliva and, therefore, the enclosed atorvastatin (1%) was fully released in less than 24 h. This result was similar to a study by Ahmed et al., who also observed a complete release from methylcellulose with 1.2% atorvastatin within a day [51]. In this regard, alginate particles demonstrated superior stability for at least 7 days corresponding to a 10-fold higher dissolution time compared to conventional gel. The high stability of alginate can be attributed to the slow exchange of sodium ions from the surrounding medium for calcium ions in the particles during the degradation process [52]. Dewangan et al. previously encapsulated atorvastatin within alginate microspheres and confirmed through Fourier-transform infrared spectroscopy that alginate acts as a neutral carrier, showing no interaction with the loaded drug. This property is critical for compatibility and safety in future clinical applications [53].

Changing the polymer concentration has previously been reported as an effective strategy to improve drug entrapment. For example, Reddy and Nagabhushanam prepared atorvastatin-loaded and alginate-based hydrogel beads using ionic gelation. Authors stated that low alginate concentrations (1–2%) resulted in a loose polymer network, leading to a higher fluid absorption and a higher swelling rate. However, a loose matrix also accounted for the facilitated drug diffusion, which increased the release rate. In contrast, raising the alginate amount to 3% decelerated drug release [54]. Although particles examined in the aforementioned study were much larger (480–1132 µm), we observed a similar trend in the release profile of our particles (<400 µm). Here, the higher concentration of polymer accounted for the prolonged time interval required to release 50% of bound atorvastatin (t_50%_). In the group of larger particles (200–400 µm), a 2-fold release delay was achieved by increasing alginate content from 2% to 3% (1.00 h vs. 2.58 h). Further polymer enrichment to 4% slightly shortened the t_50%_ interval (1.94 h), while concentrations above 5% produced a highly viscous solution that could not be properly dispersed. Considering the smaller group (10–200 µm), a similar release profile was observed for polymer loads of 3% and higher. Although low alginate concentration (2%) led to the longest t_50%_ interval (4.15 h) in the smaller particle group, prolongation was probably linked to the observed agglomeration of particles that could have prevented drug diffusion. Agglomeration occurs as a common reaction in small particles with low polymer contents (2%) [55,56]. Adopting the Korsmeyer–Peppas model revealed that in the large (2%) group, drug release followed a Fickian diffusion pattern, where the low polymer matrix density enabled rapid diffusion along concentration gradient. Due to clumping, the small group was marked by a significant release delay, probably leading to the non-Fickian transport mechanism, which is a combined action of passive diffusion and polymer relaxation. In contrast, higher polymer content increased the network density and shifted the release mechanism from passive diffusion to Super Case II transport in both groups, indicating that polymer erosion rather than mere diffusion was the dominant factor for delayed drug release. This transition is particularly important for drug formulations aiming for a controlled and prolonged release.

In fact, the most critical factor for a continuous drug release over several days was the therapeutic load in the particles. To prevent agglomeration and its possible impact on drug release, only particles in range of 200–400 µm were examined. The polymer load was set to 2% to provide more area for drug incorporation due to the raised drug dosage. Increasing the atorvastatin concentration from 1% to 4% extended the t_50%_ interval from 1.00 h to 10.83 h and shifted the release mechanism from passive diffusion to Case II transport. Thus, polymer swelling and relaxation were the primary driving forces for the delayed drug expulsion. Traces of released atorvastatin could be detected up to 123.35 ± 3.55 h of incubation, indicating an effective release interval of ca. 5 days, which was a 7-fold improvement over conventional methylcellulose carriers.

In addition, a delayed release could be achieved by introducing a covalent cross-linking agent to increase the mechanical strength and stabilize the alginate matrix. Numerous methods for covalent cross-linking have already been described in the literature [33]. Divinyl sulfone has been well established as a coupling reagent in the cross-linking of hyaluronic acid and is used clinically for this purpose [57,58,59]. The formation of reactive radicals in divinyl sulfone is induced by an alkaline medium, such as sodium hydroxide. DVS then reacts with the hydroxyl groups of hyaluronic acid to form a network amongst the polymer chains. The alginate scaffold also provides a substantial proportion of reactive functional groups such as carboxyl and hydroxyl groups, which are highly beneficial for a successful cross-linking reaction [60]. DVS-modified hyaluronic acid features greater mechanical stabilization, an extended molecular degradation period, and maintained in vitro biocompatibility at DVS concentrations up to 50 mM [61]. However, DVS has not yet been used to stabilize alginate particles. In 2012, Yu and Chau investigated the impact of DVS on various polymers containing hydroxyl groups, including alginate, by mixing the polymers with 0.1 M NaOH and DVS. The molecular structure was examined using proton nuclear magnetic resonance spectroscopy. The presence of vinyl groups was detected in the spectra obtained in all evaluated polymers, thereby confirming the established cross-linking characteristics [62]. Introduction of DVS to our preparation method led to a significant prolongation of t_50%_ interval by ~3 h, indicating an improved release control; however, the total release interval was not affected by DVS. This observation might result from the more rigid structure of DVS-modified particles, causing an initially stronger resistance against hydration. Drug transport has also shifted from Case II to Super Case II behavior after the addition of DVS (*n* = 0.91 vs. *n* = 1.08, respectively). We suggest that erosion of the polymer facilitated drug release once swelling started, while the particles without DVS cross-linking were less affected by degradation. Hence, the DVS cross-linking delayed the initial release by enhancing the mechanical stability of the matrix, whereas t_99.5%_ remained unaffected due to the accelerated drug release caused by additional polymer degradation. Follow-up studies and optimization of the cross-linking protocol are required to assess the benefits of cross-linking, including the testing of different DVS concentrations or other biocompatible covalent cross-linkers.

From a clinical point of view, alginate microparticles are considered suitable for use in dentistry, as their composition allows application in conventional syringe systems. Particles of different sizes can be selected to fit into gingival pockets (ca. 4 mm × 0.5 mm), thereby allowing a patient-specific application [15,63]. The 1% atorvastatin content used in our system is similar to the previously reported clinical administration of MC gel, which showed significant improvements in clinical parameters in periodontitis patients after a single dose [27]. Another in vivo study reported significant alveolar bone healing and decreased inflammatory effects in periodontitis rats after treatment with 2% atorvastatin delivered by a chitosan gel [64]. In a clinical context, systemic doses of atorvastatin ranged from 10 to 80 mg/day. Lower doses between 1 µg to 50 mg/body weight were reported for local statin delivery [65]. In the case of severe periodontal defects, the pocket depth could extend from 4 mm to 12 mm, which would be sufficient for the application of 250 mg alginate microparticles. In the case of 2% atorvastatin load, ca. 5 mg of the drug could be released at the application site. This would be within the range of therapeutic concentrations commonly used in various in vivo studies [65]. While oral statins have several side effects, animal studies have shown that locally applied atorvastatin is not harmful to membranes and soft tissues [66,67]. However, atorvastatin has not been widely used in clinical practice. Future in vivo and clinical studies are essential to define the optimal dose for periodontal delivery.

Our in vitro study provided initial indications on the functionality of the alginate-based drug carrier for the dental application of atorvastatin. However, in vitro evidence is limited and cannot fully reflect the complex interactions in the living organism or the conditions in a gingival pocket. Hence, the stability and degradation of the alginate particles must be investigated experimentally in further studies. Besides intrinsic properties that determine release profiles, such as particle size, drug load, polymer content, and inclusion of additional stabilizers, external factors can also be of critical importance. For example, alginate polymer systems are subjected to complex processes upon the addition of the eluent, with temperature and pH playing a prominent role [68,69]. Calcium alginate is known to swell under neutral and alkaline conditions and drugs can diffuse out more quickly [70]. Less swelling is observed under acidic conditions [71]. In our study, artificial saliva with neutral pH (7) was used. Thus, swelling of the alginate particles was observed in all tests and could explain the rapid release at the beginning of the measurement period in almost all examined samples. However, the effect could be different in the case of future clinical applications, as the pH of the sulcus fluid varies in a periodontal pocket. While an average pH value of 6.5 can be measured on healthy gingiva, this can drop to 5.4 in cases of gingival inflammation [72]. Therefore, subsequent studies should emphasize the release rates under different pH conditions to ensure a proper dosage. Further in vivo validation is also necessary to ensure the efficacy and tolerability of our system.

## 5. Conclusions

In this in vitro work, we established an optimized production method of alginate-based microparticles using an ultrasonic processor and evaluated the release of enclosed atorvastatin from different particle formulations. The microparticles exhibited superior drug retention and stability compared to conventional methylcellulose gel. Inclusion of divinyl sulfone to the formulation significantly stabilized atorvastatin diffusion at the early release phase. The low alginate content (2%) accounted for the frequent agglomeration between small particles that was absent in the corresponding large group. The 3% polymer content was optimal for both groups, allowing twice as long release than that in the methylcellulose carrier. The most profound impact on decelerating drug release was achieved by increasing the drug load to 2–4%, which maintained the controlled release profile over several days. In the future, alginate microparticles could be a valuable alternative to existing drug carriers in the field of periodontology. However, further in vitro and in vivo validation studies are necessary to evaluate the cytotoxic and therapeutic effects.

## Figures and Tables

**Table 1:** Composition of artificial saliva for the analysis of in vitro release kinetics.

Reagents	Weight per 100 g
Potassium chloride (Merck, Darmstadt, Germany)	0.12 g
Sodium chloride (Merck, Darmstadt, Germany)	0.085 g
Disodium hydrogen phosphate (Merck, Darmstadt, Germany)	0.25 g
Purified water	99.545 g

**Table 2:** Technical characteristics of HPLC analysis.

Parameter	Setting
Column	Knauer Eurospher 100-5 C18, 250 × 4 mm
Mobile phase	Methanol /phosphate buffered saline (pH 4.0; 70%/30% *v*/*v*)
Column temperature	62 °C
Flow rate	1 mL/min
Detection wavelength	UV 248 nm
Injected sample volume	20 µL
Retention time	2 min
Calibration standards	0.1, 1, 5, 10, 20, and 50 mg/L atorvastatin dissolved in methanol/purified water (80%/20% *v*/*v*)
Calibrated measuring range	0.1–50 µg/mL

**Table 3:** Time required for 50% (t_50%_) and 99.5% (t_99.5%_) drug release and dissolution time (DT) for various formulations based on methylcellulose (MC) or alginate particles produced with either a dental scaler (DS) or an ultrasonic processor (UP) with or without the addition of divinyl sulfone (DVS). The statistical comparison of t_50%_ and t_99.5%_ were conducted within groups that were delineated by color coding (yellow, green, orange). Fickian diffusion is indicated for *n* ≤ 0.45, non-Fickian diffusion is indicated for 0.45 < *n* < 0.89, Case II transport is indicated for *n* = 0.89, and Super Case II transport is indicated for *n* > 0.89.

Formulation					Diffusion	t_50%_ ± SD [h]	t_99.5%_ ± SD [h]	DT [h]
Method	Atorvastatin content [%]	Particle size [µm]	Alginate content [%]	Cross-linker	Exponent *n*			
MC	1	-	2	no	1.09	1.78 ± 0.18	12.5	17
DS	1	10–200	2	no	0.21	0.45 ^†^	169 ^†^	>177
DS	1	200–400	2	no	1.57	3.08 ± 0.39	26.44 ± 6.66	>177
UP	1	10–200	2	no	0.55	4.15 ± 0.98 *^†^	71.35 ± 19.86 ^†^	>177
UP	1	10–200	3	no	1.23	2.24 ± 0.42	30.82 ± 10.88	>177
UP	1	10–200	4	no	1.06	1.91 ± 0.21	19.09 ± 8.38	>177
UP	1	200–400	2	no	0.37	1.00 ± 0.27	17.54 ± 8.09	>177
UP	1	200–400	3	no	1.46	2.58 ± 0.90 *	24.10 ± 8.62	>177
UP	1	200–400	4	no	1.52	1.94 ± 0.68	12.65 ± 5.11	>177
UP	2	200–400	2	no	0.91	4.97 ± 1.32	91.26 ± 11.19	>177
UP	2	200–400	2	DVS	1.08	7.52 ± 3.08 *	95.84 ± 16.07	>177
UP	4	200–400	2	no	0.90	10.83 ± 1.07	123.35 ± 3.55	>177

* Significantly prolonged drug release compared within coloured group (*p* < 0.05). ^†^ Increased agglomeration of particles.

## Data Availability

The original contributions presented in this study are included in the article. Further inquiries can be directed to the corresponding author.

## References

[1] Trindade D., Carvalho R., Machado V., Chambrone L., Mendes J.J., Botelho J. (2023). Prevalence of periodontitis in dentate people between 2011 and 2020: A systematic review and meta-analysis of epidemiological studies. J. Clin. Periodontol..

[2] Wolf H.F., Rateitschak E.M., Rateitschak K.-H., Schroeder H.E. (2012). Einleitung. Parodontologie. Farbatlanten der Zahnmedizin 1, 3., Vollständig Überarbeitete und Erweiterte Auflage.

[3] Wei Y., Deng Y., Ma S., Ran M., Jia Y., Meng J., Han F., Gou J., Yin T., He H. (2021). Local drug delivery systems as therapeutic strategies against periodontitis: A systematic review. J. Control. Release.

[4] Lalla E., Papapanou P.N. (2011). Diabetes mellitus and periodontitis: A tale of two common interrelated diseases. Nat. Rev. Endocrinol..

[5] Sanz M., Marco Del Castillo A., Jepsen S., Gonzalez-Juanatey J.R., D’Aiuto F., Bouchard P., Chapple I., Dietrich T., Gotsman I., Graziani F. (2020). Periodontitis and cardiovascular diseases: Consensus report. J. Clin. Periodontol..

[6] Smiley C.J., Tracy S.L., Abt E., Michalowicz B.S., John M.T., Gunsolley J., Cobb C.M., Rossmann J., Harrel S.K., Forrest J.L. (2015). Evidence-based clinical practice guideline on the nonsurgical treatment of chronic periodontitis by means of scaling and root planing with or without adjuncts. J. Am. Dent. Assoc..

[7] Sanderink R.B.A., Renggli H.H., Saxer U.P. (2022). Orale Präventivmedizin. Eine Interdisziplinäre Herausforderung, 1. Auflage.

[8] Isola G., Polizzi A., Santonocito S., Dalessandri D., Migliorati M., Indelicato F. (2021). New Frontiers on Adjuvants Drug Strategies and Treatments in Periodontitis. Sci. Pharm..

[9] Jain N., Jain G.K., Javed S., Iqbal Z., Talegaonkar S., Ahmad F.J., Khar R.K. (2008). Recent approaches for the treatment of periodontitis. Drug Discov. Today.

[10] Tønnesen H.H., Karlsen J. (2002). Alginate in drug delivery systems. Drug Dev. Ind. Pharm..

[11] Subbarao K.C., Nattuthurai G.S., Sundararajan S.K., Sujith I., Joseph J., Syedshah Y.P. (2019). Gingival Crevicular Fluid: An Overview. J. Pharm. Bioallied Sci..

[12] Ghavami-Lahiji M., Shafiei F., Najafi F., Erfan M. (2019). Drug-loaded polymeric films as a promising tool for the treatment of periodontitis. J. Drug Deliv. Sci. Technol..

[13] Zamani M., Morshed M., Varshosaz J., Jannesari M. (2010). Controlled release of metronidazole benzoate from poly epsilon-caprolactone electrospun nanofibers for periodontal diseases. Eur. J. Pharm. Biopharm..

[14] Joshi D., Garg T., Goyal A.K., Rath G. (2016). Advanced drug delivery approaches against periodontitis. Drug Deliv..

[15] Scholz M., Reske T., Böhmer F., Hornung A., Grabow N., Lang H. (2017). In vitro chlorhexidine release from alginate based microbeads for periodontal therapy. PLoS ONE.

[16] Swain S., Behera A., Beg S., Patra C.N., Dinda S.C., Sruti J., Rao M.E.B. (2012). Modified alginate beads for mucoadhesive drug delivery system: An updated review of patents. Recent Pat. Drug Deliv. Formul..

[17] Tahamtan S., Shirban F., Bagherniya M., Johnston T.P., Sahebkar A. (2020). The effects of statins on dental and oral health: A review of preclinical and clinical studies. J. Transl. Med..

[18] German C.A., Liao J.K. (2023). Understanding the molecular mechanisms of statin pleiotropic effects. Arch. Toxicol..

[19] Bertl K., Parllaku A., Pandis N., Buhlin K., Klinge B., Stavropoulos A. (2017). The effect of local and systemic statin use as an adjunct to non-surgical and surgical periodontal therapy-A systematic review and meta-analysis. J. Dent..

[20] Bertl K., Steiner I., Pandis N., Buhlin K., Klinge B., Stavropoulos A. (2018). Statins in nonsurgical and surgical periodontal therapy. A systematic review and meta-analysis of preclinical in vivo trials. J. Periodontal Res..

[21] Kumari M., Martande S.S., Pradeep A.R., Naik S.B. (2016). Efficacy of Subgingivally Delivered 1.2% Atorvastatin in the Treatment of Chronic Periodontitis in Patients with Type 2 Diabetes Mellitus: A Randomized Controlled Clinical Trial. J. Periodontol..

[22] Shah S.R., Werlang C.A., Kasper F.K., Mikos A.G. (2015). Novel applications of statins for bone regeneration. Natl. Sci. Rev..

[23] Shirke P.Y., Kolte A.P., Kolte R.A., Bawanakar P.V. (2019). Evaluation of the clinical efficacy of 1.2% atorvastatin in the treatment of periodontal intraosseous defects by CBCT: A randomized controlled clinical trial. J. Dent. Res. Dent. Clin. Dent. Prospect..

[24] Bonetti L., de Nardo L., Farè S. (2023). Crosslinking strategies in modulating methylcellulose hydrogel properties. Soft Matter.

[25] Fu Y., Kao W.J. (2010). Drug release kinetics and transport mechanisms of non-degradable and degradable polymeric delivery systems. Expert Opin. Drug Deliv..

[26] Contri R.V., Gazzi R.P., Pohlmann A.R., Guterres S.S., Frank L.A., Talevi A. (2022). Drug Release from Pharmaceutical Nanocarriers. The ADME Encyclopedia.

[27] Pradeep A.R., Kumari M., Rao N.S., Martande S.S., Naik S.B. (2013). Clinical efficacy of subgingivally delivered 1.2% atorvastatin in chronic periodontitis: A randomized controlled clinical trial. J. Periodontol..

[28] Shimojo A.A.M., Pires A.M.B., Lichy R., Santana M.H.A. (2015). The Performance of Crosslinking with Divinyl Sulfone as Controlled by the Interplay Between the Chemical Modification and Conformation of Hyaluronic Acid. J. Braz. Chem. Soc..

[29] Kumar A. (2016). Supermacroporous Cryogels.

[30] Goodson J.M., Offenbacher S., Farr D.H., Hogan P.E. (1985). Periodontal disease treatment by local drug delivery. J. Periodontol..

[31] Soskolne W.A. (1997). Subgingival delivery of therapeutic agents in the treatment of periodontal diseases. Crit. Rev. Oral Biol. Med..

[32] Rajeshwari H.R., Dhamecha D., Jagwani S., Rao M., Jadhav K., Shaikh S., Puzhankara L., Jalalpure S. (2019). Local drug delivery systems in the management of periodontitis: A scientific review. J. Control. Release.

[33] Aguero L., Alpdagtas S., Ilhan E., Zaldivar-Silva D., Gunduz O. (2021). Functional role of crosslinking in alginate scaffold for drug delivery and tissue engineering: A review. Eur. Polym. J..

[34] Fernandes G., Wang C., Yuan X., Liu Z., Dziak R., Yang S. (2016). Combination of Controlled Release Platelet-Rich Plasma Alginate Beads and Bone Morphogenetic Protein-2 Genetically Modified Mesenchymal Stem Cells for Bone Regeneration. J. Periodontol..

[35] Kaigler D., Silva E.A., Mooney D.J. (2013). Guided bone regeneration using injectable vascular endothelial growth factor delivery gel. J. Periodontol..

[36] Sands R.W., Verbeke C.S., Ouhara K., Silva E.A., Hsiong S., Kawai T., Mooney D. (2020). Tuning cytokines enriches dendritic cells and regulatory T cells in the periodontium. J. Periodontol..

[37] Chen X., Wu G., Feng Z., Dong Y., Zhou W., Li B., Bai S., Zhao Y. (2016). Advanced biomaterials and their potential applications in the treatment of periodontal disease. Crit. Rev. Biotechnol..

[38] Chen L., Shen R., Komasa S., Xue Y., Jin B., Hou Y., Okazaki J., Gao J. (2017). Drug-Loadable Calcium Alginate Hydrogel System for Use in Oral Bone Tissue Repair. Int. J. Mol. Sci..

[39] Lopes M., Abrahim B., Veiga F., Seiça R., Cabral L.M., Arnaud P., Andrade J.C., Ribeiro A.J. (2017). Preparation methods and applications behind alginate-based particles. Expert Opin. Drug Deliv..

[40] Campos E., Branquinho J., Carreira A.S., Carvalho A., Coimbra P., Ferreira P., Gil M.H. (2013). Designing polymeric microparticles for biomedical and industrial applications. Eur. Polym. J..

[41] Łętocha A., Miastkowska M., Sikora E. (2022). Preparation and Characteristics of Alginate Microparticles for Food, Pharmaceutical and Cosmetic Applications. Polymers.

[42] Dalmoro A., d’Amore M., Barba A.A. (2013). Droplet size prediction in the production of drug delivery microsystems by ultrasonic atomization. Transl. Med. @ UniSa.

[43] Patel M.K., Praveen B., Sahoo H.K., Patel B., Kumar A., Singh M., Nayak M.K., Rajan P. (2017). An advance air-induced air-assisted electrostatic nozzle with enhanced performance. Comput. Electron. Agric..

[44] Zhang C., Grossier R., Candoni N., Veesler S. (2021). Preparation of alginate hydrogel microparticles by gelation introducing cross-linkers using droplet-based microfluidics: A review of methods. Biomater. Res..

[45] Dalmoro A., Barba A.A., Lamberti G., d’Amore M. (2012). Intensifying the microencapsulation process: Ultrasonic atomization as an innovative approach. Eur. J. Pharm. Biopharm..

[46] Naidu H., Kahraman O., Feng H. (2022). Novel applications of ultrasonic atomization in the manufacturing of fine chemicals, pharmaceuticals, and medical devices. Ultrason. Sonochem..

[47] Cascone S., Lamberti G., Titomanlio G., Barba A.A., d’Amore M. (2012). Microencapsulation effectiveness of small active molecules in biopolymer by ultrasonic atomization technique. Drug Dev. Ind. Pharm..

[48] Kumari M., Martande S.S., Pradeep A.R. (2017). Subgingivally delivered 1.2% atorvastatin in the treatment of chronic periodontitis among smokers: A randomized, controlled clinical trial. J. Investig. Clin. Dent..

[49] Pradeep A.R., Kanoriya D., Singhal S., Garg V., Manohar B., Chatterjee A. (2017). Comparative evaluation of subgingivally delivered 1% alendronate versus 1.2% atorvastatin gel in treatment of chronic periodontitis: A randomized placebo-controlled clinical trial. J. Investig. Clin. Dent..

[50] Wüstenberg T. (2013). Cellulose und Cellulosederivate. Grundlagen, Wirkungen und Applikationen, 1. Aufl..

[51] Ahmed M.G., Choudhari R., Acharya A. (2015). Formulation and Evaluation of In situ Gel of Atorvastatin for the Treatment of Periodontitis. RGUHS J. Pharm. Sci..

[52] Rahimi M., Noruzi E.B., Sheykhsaran E., Ebadi B., Kariminezhad Z., Molaparast M., Mehrabani M.G., Mehramouz B., Yousefi M., Ahmadi R. (2020). Carbohydrate polymer-based silver nanocomposites: Recent progress in the antimicrobial wound dressings. Carbohydr. Polym..

[53] Dewangan H.K., Sharma A., Mishra A., Singour P. (2021). Mucoadhesive Microspheres of Atorvastatin Calcium: Rational Design, Evaluation and Enhancement of Bioavailability. IJPER.

[54] Reddy K.V.R., Nagabhushanam M.V. (2019). Preparation of gastro retentive mucoadhesive beads for atorvastatin by using ionic gelation method with divalence and trivalence curing agents and their characterization studies. IJPSR.

[55] Mackin-Mohamour A.-R.S., Budzinski J., Bastogne T., Roques-Carmes T., Sadtler V., Marchal P., Sapin-Minet A., Parent M. (2024). Agile quality-by-design development of alginate microparticles for encapsulation of hydrophilic drug. Colloids Surf. A Physicochem. Eng. Asp..

[56] Rajinikanth P.S., Sankar C., Mishra B. (2003). Sodium alginate microspheres of metoprolol tartrate for intranasal systemic delivery: Development and evaluation. Drug Deliv..

[57] Ibrahim S., Kang Q.K., Ramamurthi A. (2010). The impact of hyaluronic acid oligomer content on physical, mechanical, and biologic properties of divinyl sulfone-crosslinked hyaluronic acid hydrogels. J. Biomed. Mater. Res. Part A.

[58] Oh E.J., Kang S.-W., Kim B.-S., Jiang G., Cho I.H., Hahn S.K. (2008). Control of the molecular degradation of hyaluronic acid hydrogels for tissue augmentation. J. Biomed. Mater. Res. Part A.

[59] Sannino A., Madaghiele M., Conversano F., Mele G., Maffezzoli A., Netti P.A., Ambrosio L., Nicolais L. (2004). Cellulose derivative-hyaluronic acid-based microporous hydrogels cross-linked through divinyl sulfone (DVS) to modulate equilibrium sorption capacity and network stability. Biomacromolecules.

[60] Yang J.-S., Xie Y.-J., He W. (2011). Research progress on chemical modification of alginate: A review. Carbohydr. Polym..

[61] Lai J.-Y. (2014). Relationship between structure and cytocompatibility of divinyl sulfone cross-linked hyaluronic acid. Carbohydr. Polym..

[62] Yu Y., Chau Y. (2012). One-step “click” method for generating vinyl sulfone groups on hydroxyl-containing water-soluble polymers. Biomacromolecules.

[63] Al Shayeb K.N.A., Turner W., Gillam D.G. (2014). Periodontal probing: A review. Prim. Dent. J..

[64] Özdoğan A.I., İlarslan Y.D., Kösemehmetoğlu K., Akca G., Kutlu H.B., Comerdov E., Iskit A.B., Şenel S. (2018). In vivo evaluation of chitosan based local delivery systems for atorvastatin in treatment of periodontitis. Int. J. Pharm..

[65] Oryan A., Kamali A., Moshiri A. (2015). Potential mechanisms and applications of statins on osteogenesis: Current modalities, conflicts and future directions. J. Control. Release.

[66] de Miranda-Filho F.V., Barbosa S., Panigali O.A., Silva M.C., Da Costa M.G., Da Flores F.S., Ervolino E., Theodoro L.H., Magro-Filho O., Faverani L.P. (2024). Effect of local and systemic administration of atorvastatin for improving bone healing on critical defects. Braz. Dent. J..

[67] Shahrezaee M., Oryan A., Bastami F., Hosseinpour S., Shahrezaee M.H., Kamali A. (2018). Comparative impact of systemic delivery of atorvastatin, simvastatin, and lovastatin on bone mineral density of the ovariectomized rats. Endocrine.

[68] Langer R., Peppas N. (1983). Chemical and Physical Structure of Polymers as Carriers for Controlled Release of Bioactive Agents: A Review. J. Macromol. Sci. Part C.

[69] Arifin D.Y., Lee L.Y., Wang C.-H. (2006). Mathematical modeling and simulation of drug release from microspheres: Implications to drug delivery systems. Adv. Drug Deliv. Rev..

[70] Shi J., Alves N.M., Mano J.F. (2006). Drug release of pH/temperature-responsive calcium alginate/poly(N-isopropylacrylamide) semi-IPN beads. Macromol. Biosci..

[71] Bajpai S.K., Sharma S. (2004). Investigation of swelling/degradation behaviour of alginate beads crosslinked with Ca^2+^ and Ba^2+^ ions. React. Funct. Polym..

[72] Dakshayani B., Thamilselvi D., Rosaline H., Kandaswamy D. (2020). Evaluation of pH of Gingival Crevicular Fluid as a Local Factor in Etiology of Cervical Lesions. Int. J. Dent. Sci. Res..

